# Torsion of a congenital human tail in a Japanese Infant: A case report

**DOI:** 10.1016/j.jpra.2026.01.026

**Published:** 2026-01-24

**Authors:** Yoshie Sasaki, Ken-ichiro Konishi, Kei Nakano, Akira Nishi, Junko Hirato, Charles M. Malata, Akito Hamajima

**Affiliations:** a Department of Plastic Surgery, Gunma Children's Medical Center, Shibukawa-shi, Gunma, Japan; b Department of Surgery, Gunma Children's Medical Center, Shibukawa-shi, Gunma, Japan; c Department of Pathology, Public Tomioka General Hospital, Tomioka-shi, Gunma, Japan; d Department of Plastic Surgery, Cambridge University Hospitals NHS Foundation Trust, Cambridge CB2 0QQ, UK; e Anglia Ruskin University School of Medicine, Chelmsford and Cambridge, UK

**Keywords:** Human tail, Torsion, Neonatal surgery, Spinal dysraphism, Emergency surgery

## Abstract

The optimal timing of surgical intervention for a human tail remains unclear. We report a 2-month-old male infant with a tail-like structure in the sacral region, lacking continuity with the spinal cord. Although elective surgery was scheduled at 3 months of age, vascular compromise due to torsion developed on postnatal day 60, requiring emergency excision under local anesthesia. The postoperative course was uneventful, with preserved limb function and no complications at follow-up. This rare case highlights torsion as a potential complication and supports early surgical intervention, even during the neonatal period, once imaging confirms the absence of spinal cord involvement and spinal dysraphism, particularly in cases with a constricted base.

## Introduction

The human tail is a rare benign congenital anomaly characterized by a tail-like structure in the lumbosacrococcygeal region.^1^^,^^2^ Surgical management of the human tail requires careful consideration of three key factors: continuity between the tail and spinal cord, the presence of spinal dysraphism, and the necessity and optimal timing of neurosurgical intervention.^3^^,^^4^ Despite these considerations, the optimal timing for surgical intervention remains unclear. Although the condition is diagnosed at birth and frequently requires surgical treatment, there is no consensus on the ideal timing of surgery, even when early intervention is feasible.

We report a case of a human tail requiring early surgical intervention due to vascular compromise caused by torsion at the base during the preoperative waiting period. As torsion of the human tail is an extremely rare complication, this case provides important evidence in support of early surgical intervention.

## Case report

The patient was a 2-month-old male infant born at 38 weeks of gestation via normal vaginal delivery, with a birth weight of 3.310 g. At birth, a midline mass measuring 1 cm in diameter and 7.5 cm in length was observed in the sacral region. The patient was referred to our hospital on postnatal day 40.

**Figure 1:**
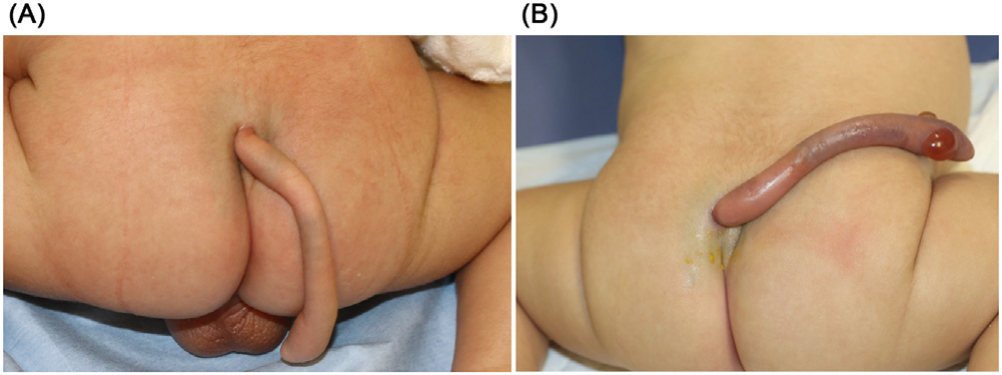
Clinical appearance of the lesion (a true human tail—Tojima and Yamada type IIa). (A) A pedunculated cylindrical structure measuring 1 cm in diameter and 9 cm in length, with a constricted base, was observed in the midline cranial region of the gluteal cleft. The structure appeared soft and elastic, with no palpable firm tissue at or within the base. The surface was covered with normal skin tissue. It exhibited no movement (spontaneous or otherwise). No associated spinal dysraphism or other congenital malformations were observed. (B) The human tail is twisted at its constricted base. The entire structure appeared mildly swollen. The skin surface exhibited dark purplish discoloration and blister formation.

**Figure 2:**
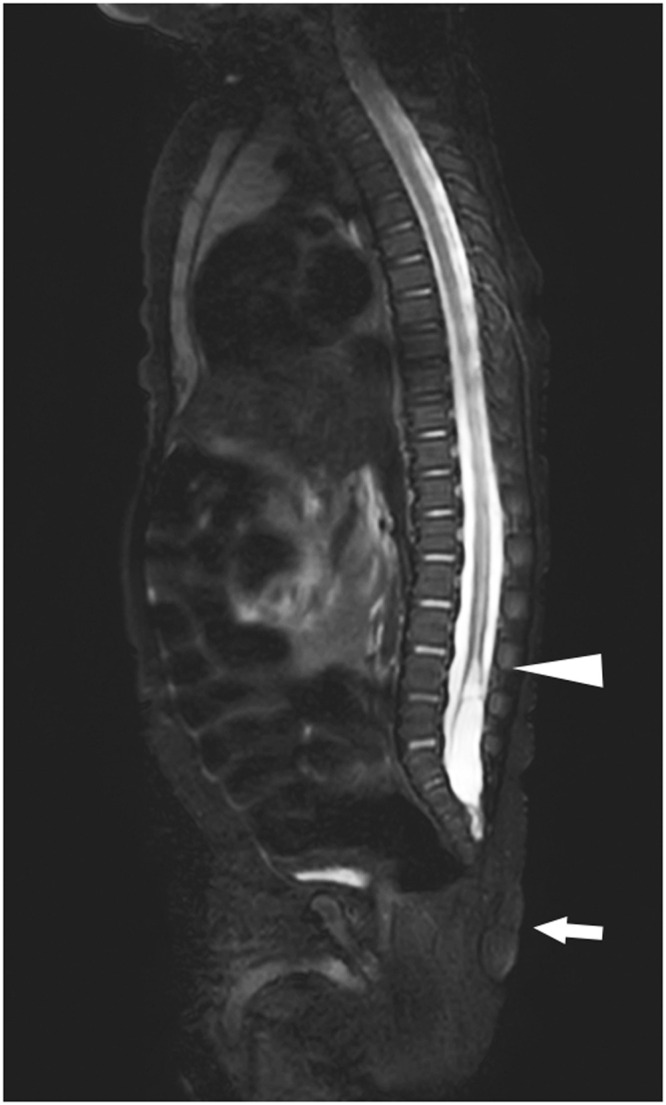
Spinal MRI findings (T2-weighted images with fat suppression). No continuity was observed between the human tail and the spinal canal or its internal structures. The arrow indicates the location of the tail. A low-lying conus medullaris is visible at the L3 level (arrowhead), with cystic expansion at the terminal portion of the spinal cord.

On examination, a cylindrical structure measuring 1 cm in diameter and 9 cm in length with a constricted base was observed at the cranial aspect of the gluteal cleft. On palpation, the structure was soft and elastic, with no palpable firm tissue within or at its base (Figure 1A). The surface was covered with normal skin and ectopic Mongolian spots. No voluntary movements were observed. Motor function in both lower limbs was normal, and diaper changes were required every 2–3 h, which was consistent with age-appropriate norms. Based on these features, a diagnosis of a human tail was made. No other congenital anomalies were observed. Ultrasound and magnetic resonance imaging (MRI) revealed a low-lying conus medullaris at the L3 level and a terminal myelocystocele; however, no continuity was observed between the tail and spinal canal (Figure 2). Elective surgery was scheduled at 3 months of age to minimize interference with developmental milestones, including head control. However, on postnatal day 60, the patient returned to our department with tail discoloration and increased irritability. Examination revealed mild swelling, dark purplish discoloration, and blister formation (Figure 1B). The structure was not tender.

**Figure 3:**
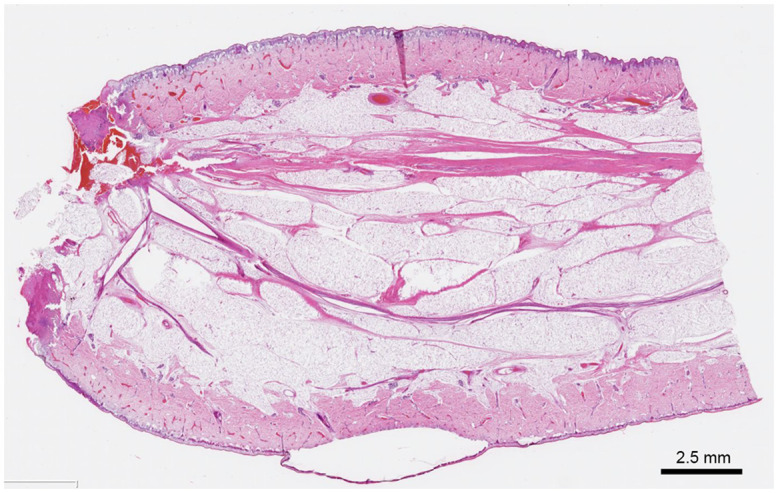
Histopathological Findings. Hematoxylin and Eosin (HE) staining (longitudinal section). On HE staining of the root of the human tail revealed cord-like fibrous connective tissue. The lesion was covered by normal skin tissue, with a subepidermal blister present in part of the epidermis. Adipose tissue continuous with subcutaneous fat and thick peripheral nerves was observed.

Vascular compromise due to torsion at the base of the tail was suspected, prompting emergency ligation and excision on the same day. The base was ligated with 5-0 Nylon and excised under local anesthesia. The postoperative course was uneventful, with no signs of infection, and the patient was discharged the next day. Histological examination revealed that the lesion was covered by normal skin with focal subepidermal blister formation. The central portion consisted of adipose tissue continuous with the subcutaneous layer. In contrast, the base contained fibrous connective tissue strands (Figure 3, Supplementary Figure 1A) and thin peripheral nerve fiber bundles (Supplementary Figure 1B). Notably, no spinal cord tissue, cartilage, bone, or skeletal muscle was identified throughout the specimen, findings that are consistent with the presence of a human tail. At 3 months of age (38 days postoperatively), the motor function in both lower limbs remained intact, and no abnormalities were observed at the site of excision.

## Discussion

We report a case of a human tail complicated by vascular compromise due to torsion at its base while waiting for elective surgery. Given the risks of necrosis and infection, which may become life-threatening, we recommend early surgical intervention, including during the neonatal period. Criteria that may support neonatal surgery in human tail cases include a constricted base, lack of continuity with the spinal cord, and morphology prone to torsion.

Surgical management of a human tail requires evaluation of three key factors: continuity with the spinal cord, presence of spinal dysraphism, and necessity and timing of neurosurgical intervention.^5^, ^6^, ^7^ In our case, the tail had no continuity with the spinal cord, no associated spinal dysraphism, and no indication for neurosurgical intervention. Although surgery was initially scheduled for 3 months of age, torsion necessitated emergency ligation and excision. To preserve the option for future neurosurgical management, excision was limited to the constricted base, without deep dissection. On postoperative day 38, there were no motor deficits or postoperative complications. We advocate early surgical intervention, ideally during the neonatal period, once MRI confirms the absence of spinal cord continuity and rules out spinal dysraphism.

Early surgical intervention raises concerns regarding the risks associated with general anesthesia.^8^ For this reason, we initially delayed surgery until 3 months of age, coinciding with developmental milestones such as head control. However, emergency surgery was ultimately required because of the torsion. Torsion can lead to necrosis and wound infection, with potential systemic complications, such as cytokine-induced respiratory and circulatory effects. Given that excision can be safely performed under local anesthesia with minimal invasiveness, early surgical intervention, including during the neonatal period, should be considered to mitigate the risks associated with torsion.

The base morphology may influence the risk of torsion. To our knowledge, only one other case of human tail torsion has been reported in the literature. In that 2008 case, a 1.0 cm-wide and 5 cm-long human tail underwent torsion at 1 month of age and required emergency excision.^9^ Although the report did not describe the morphology in detail, the images suggest a constricted base, similar to our case. Given the rarity of torsion, a constricted base may be a risk factor for this condition. If torsion leads to necrosis, the resulting infection and systemic effects can be life-threatening. Therefore, early intervention should be considered in such cases.

Given the feasibility of early surgery, we propose that the management of human tails should include early MRI evaluation to assess spinal cord continuity and spinal dysraphism. In the absence of spinal cord involvement and when torsion risk is evident from morphology, surgery should be performed as early as possible, ideally within the neonatal period.

## Patient’s consent

Written informed consent was obtained from the patient’s parents. We thank the patient’s parents for their cooperation.

## Ethical approval

This study complied with the ethical principles of the Declaration of Helsinki (2013 revision) and was approved by the Ethics Committee of Gunma Children's Medical Center (approval number: GCMC2025-104).

## Declaration of competing interest

None declared.

## Appendix Supplementary materials

**Supplementary Figure 1. Histopathological Findings.** A: HE staining (cross-section). Hematoxylin and eosin (HE) staining of a cross-section approximately 3 cm from the tip. No large peripheral nerves were identified, although multiple small peripheral nerves were observed. B: Immunohistochemical staining (S100). Thin nerve fiber bundles were stained, and peripheral nerves were identified. Several similar small peripheral nerves were observed. Image, image 1 dummy alt text

## References

[1] Alashari M., Torakawa J. (1995). True tail in a newborn. Pediatr Dermatol.

[2] Tojima S., Yamada S. (2020). Classification of the "human tail": correlation between position, associated anomalies, and causes. Clin Anat.

[3] Canaz G., Akkoyun N., Emel E. (2018). A rare case of "Human tail" associated with lipomyelomeningocele and tethered cord. J Pediatr Neurosci.

[4] Dubrow T.J., Wackym P.A., Lesavoy M.A. (1988). Detailing the human tail. Ann Plast Surg.

[5] Cai C., Shi O., Shen C. (2011). Surgical treatment of a patient with human tail and multiple abnormalities of the spinal cord and column. Adv Orthop.

[6] Tubbs R.S., Malefant J., Loukas M. (2016). Enigmatic human tails: a review of their history, embryology, classification, and clinical manifestations. Clin Anat.

[7] Belzberg A.J., Myles S.T., Trevenen C.L. (1991). The human tail and spinal dysraphism. J Pediatr Surg.

[8] Disma N., O'Leary J.D., Loepke A.W. (2018). Anesthesia and the developing brain: a way forward for laboratory and clinical research. Paediatr Anaesth.

[9] Farook S., O'Kane R., Tyagi A. (2008). Tale of a human tail: case report of a torted human tail. Br J Neurosurg.

