# Supplementary figures and images for: Torsion of a congenital human tail in a Japanese Infant: A case report
Source: JPRAS Open. 2026 Jan 24;49:144–8. doi: 10.1016/j.jpra.2026.01.026 (PMC12961210; doi:10.1016/j.jpra.2026.01.026)

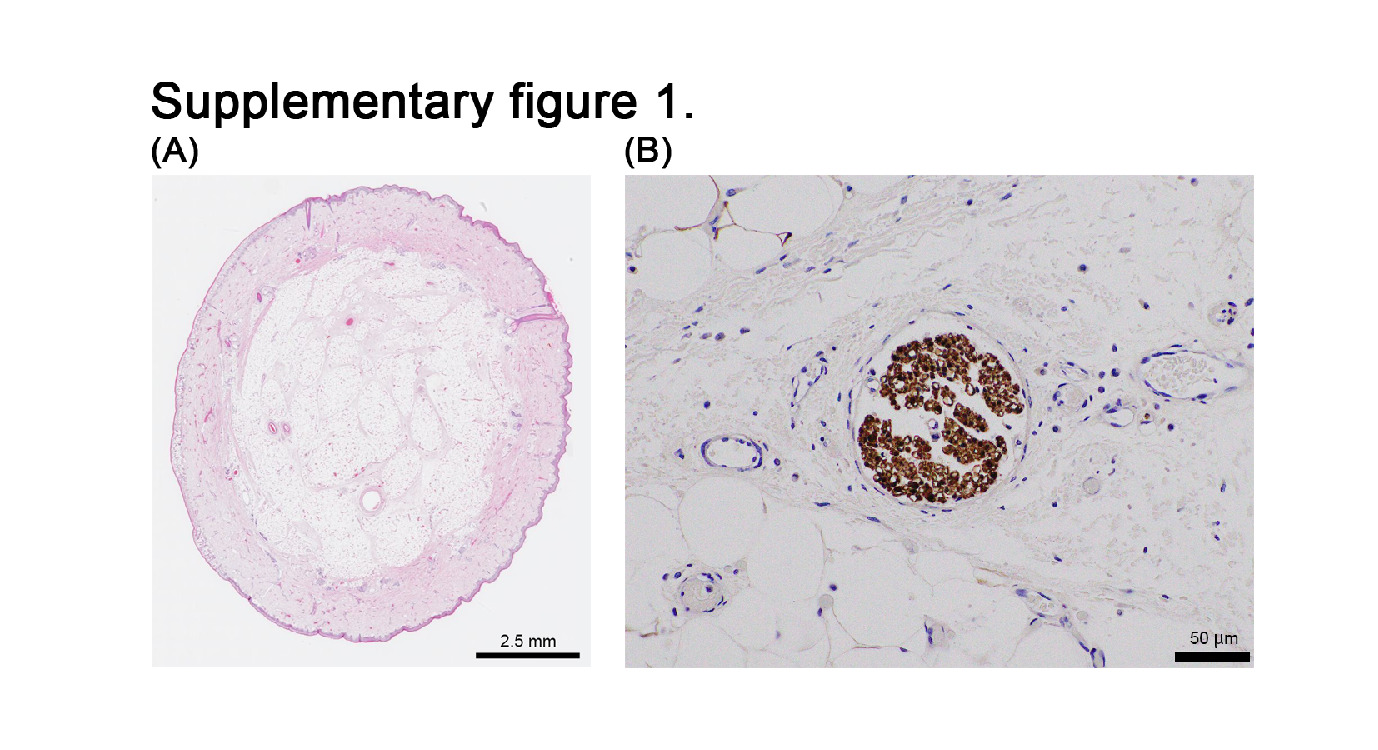

Supplement: Supplementary file 1 — Supplementary Figure 1. Histopathological Findings. A: HE staining (cross-section). Hematoxylin and eosin (HE) staining of a cross-section approximately 3 cm from the tip. No large peripheral nerves were identified, although multiple small peripheral nerves were observed. B: Immunohistochemical staining (S100). Thin nerve fiber bundles were stained, and peripheral nerves were identified. Several similar small peripheral nerves were observed. [file mmc1.jpg]
